# Monte Carlo dosimetric characterization of the IsoAid ADVANTAGE P103d brachytherapy source

**DOI:** 10.1120/jacmp.v8i2.2393

**Published:** 2007-03-20

**Authors:** Keith T. Sowards

**Affiliations:** ^1^ Department of Radiation Oncology University of Louisville, James Brown Cancer Center Louisville Kentucky

**Keywords:** Pd103, dosimetry, Monte Carlo, brachytherapy

## Abstract

For roughly 25 years, I125 and Pd103 sources have been used in the treatment of various malignant diseases such as prostate cancer. Various new sources have been marketed and produced to meet the demand for new sources to use in treatment. Recently, IsoAID LLC created the ADVANTAGE Pd103 source. Various dosimetric parameters must be determined to facilitate treatment planning using this source. Theoretical determination of dosimetric characteristics, dose rate constant, radial dose function, and anisotropy function for this new source followed the American Association of Physicists in Medicine (AAPM) Task Group 43U1 recommendations. Theoretical calculations were performed in liquid water using the PTRAN Monte Carlo code version 7.44. The radial dose function of the new source was calculated in liquid water at distances up to 10.0 cm, and the anisotropy function, at distances ranging from 0.5 cm to 7.0 cm. The anisotropy factors and anisotropy constant were derived from the anisotropy function. The results in water indicate that the dose rate constant is $0.709\pm 0.014\;\text{cGy}•h^{-1}•U^{-1}$ and that the anisotropy constant is 0.880±0.040. The dosimetric characteristics of this new source compare favorably with those of other commercially available Pd103 sources.

PACS: 87.53.Jw

## I. INTRODUCTION

For approximately 25 years, Pd103 brachytherapy sources have been produced for use in interstitial implants in various tumor sites. Sources using Pd103 are favored because their low‐energy photon emissions provide a rapid decrease in dose with increasing distance, minimizing the dose to normal tissues.

Use of ultrasound‐guided brachytherapy seed implantation for prostate cancer has increased greatly since the technique was developed in the early 1980s. The reasons for the increase are many, including patient convenience (one treatment versus many for external‐beam radiotherapy), reduced side effects as compared with radical prostatectomy, and greater cost effectiveness.^(^ ^1^ ^–^ ^4^ ^)^ With a shortage of available seeds and an increase in the number of procedures being performed nationally, several manufacturers, including IsoAid (IsoAid LLC, Port Richey, FL), have developed new Pd103 sources to meet the increasing demand.

## II. MATERIALS AND METHODS

### A. Source of Pd103

**Figure 1:**
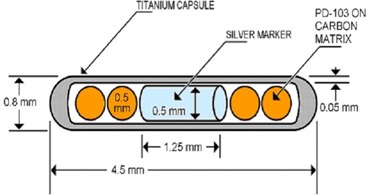
Schematic diagram of the ADVANTAGE Pd103 brachytherapy source (courtesy of IsoAid LLC). The area between the capsule and the X‐ray marker is filled with air.

The ADVANTAGE Pd103 source has a physical length of 4.5 mm and an outer diameter of 0.8 mm. To create the four active 0.5‐mm polystyrene spheres, Pd103 isotope is absorbed throughout the spheres, which are then encapsulated in a 0.05‐mm–thick titanium capsule (Fig. 1). A silver rod, 0.5 mm in diameter and 1.25 mm in length, serves as an X‐ray marker and is placed between the pair of spheres at each end of the capsule. An active length of 3.4 mm was assumed for this source during calculation of the source parameters.

### B. Monte Carlo simulation

Dose distributions for the ADVANTAGE source were calculated in liquid water using the PTRAN Monte Carlo code.^(^ ^5^ ^–^ ^7^ ^)^ Simulations were performed for up to 10 million histories divided into one hundred batches. By combining this number of histories with use of a distance‐and‐attenuation‐averaged, bounded, next‐flight point‐kerma estimator^(5)^ standard errors about the mean (67% confidence intervals) ranging from 1.5% (near the source: r<3 cm) to 5%−6% (far from the source: r>5 cm) were achieved.

One assumption made in the Monte Carlo simulation of the dose distributions around the source was that a uniform distribution of the Pd103 isotope is present within each polystyrene sphere. A density of $1.046\;g/{\text{cm}}^{3}$ was assumed for each polystyrene sphere. The X‐ray marker at the center of the source is 1.25 mm in length and 0.5 mm in diameter. The overall physical length of the source is 4.5 mm, with an outer diameter of 0.8 mm. The internal cavity of the source is filled with dry air. The PTRAN code used the Pd103 photon spectrum extracted from the Medical Internal Radiation Dosimetry (MIRD) pamphlet 10.^(6)^

The Monte Carlo–simulated dose rate constant was obtained by calculating the kerma rate to water at the reference point (1 cm, π/2) in a medium and then dividing that result by the simulated air kerma $\left(S_{K}\right)$ strength of the source. The $S_{K}$ was determined by calculating the air kerma rate at 10 cm distance and correcting for the inverse square of the distance to obtain the value at 1 cm while suppressing characteristic X‐ray production. It is understood, but impractical, to simulate at the distance that the National Institute of Standards and Technology (NIST) uses (100 cm). Such a simulation would have required extremely long run times to yield even marginal uncertainty levels. Using 10 cm as a reference yields good uncertainty at a great enough distance to fairly approximate true NIST calibration standards. In the calculations, simulations in air were performed with the titanium characteristic X‐ray production suppressed. The air kerma rate strength per unit contained activity is given in $\text{cGy}•{\text{cm}}^{2}•h^{-1}•{\text{mCi}}^{-1}$.

### C. Dosimetry technique

Characteristics of the source were determined theoretically according to Task Group Report No. 43 (TG‐43U1)^(^ ^8^ ^‐^ ^10^ ^)^ from the American Association of Physicists in Medicine (AAPM). Under that protocol, the dose distribution around a sealed brachytherapy source can be determined using the formalism

(1) $$\frac{\dot{D}(r,\theta )}{S_{K}}=\frac{\Lambda G(r,\theta )}{G(r_{o},\theta_{o})}g(r)F(r,\theta )\;\;,$$

where $S_{K}$ is the air kerma strength of the source, A is the dose rate constant at a reference point $\left(r_{0},\;\theta_{0}\right)$ or (1 cm, π/2), *G*(*r*, θ) is the geometry function, *g*(*r*) is the radial dose function, and *F*(*r*, θ) is the anisotropy function. The above quantities are thoroughly defined and discussed in great detail in TG‐43(U1) and that discussion will therefore not be repeated here.

The goal of the present project was to compare the calculated dosimetric parameters of the IsoAid ADVANTAGE Pd103 brachytherapy source with those of other commercially available Pd103 sources. The determinations were performed according to the methodology outlined in TG‐43U1^(^ ^8^ ^‐^ ^10^ ^)^ and in accordance with the AAPM recommendations for source calibration.^(11)^

## III. RESULTS

The dose rate constant, A, of the ADVANTAGE Pd103 source was determined using the equation

(2) $$\Lambda =\frac{\dot{D}(r_{0,}\theta_{0})}{S_{K}}.$$

As shown in Table 1, the Monte Carlo calculations yielded a value of $0.709\pm 0.014\;\text{cGy}•h^{-1}•U^{-1}$ in liquid water. The uncertainty of the Monte Carlo simulation was determined by combining the uncertainties of the dose rate calculated in medium and the calculated air kerma rate. The air kerma rate was obtained from the Monte Carlo data by simulating the dose rate at 1 cm in phantom and then dividing that result by the simulated dose rate at 10 cm in air with characteristic X‐ray production suppressed. The resulting value was then corrected for the effects of the inverse square law.

**Table 1:** Comparison of the dose rate constant, Λ, of the ADVANTAGE Pd103 brachytherapy source with the dose rate constants of the Theragenics Model 200, Best Industries Pd103, and NAS MED3633 sources

Source model	Reference	Method	Medium	Dose rate constant $\Lambda \;(\text{cGy}•h^{-1}•U^{-1})$ ^a^
ADVANTAGE Pd103	Present work	Monte Carlo simulation	Liquid water	0.709±0.014 ^b^
ADVANTAGE Pd103	Meigooni et al.^(12)^	Monte Carlo simulation	Liquid water	0.690±0.021 ^b^
		Monte Carlo Simulation	Solid water	0.670±0.020 ^b^
		Measured, TLD	Solid water	0.680±0.020 ^b^
Theragenics Model 200 Pd103	Williamson^(13)^	Monte Carlo simulation	Liquid water	0.680±0.020
		TG43U1	Liquid water	0.686±0.020
Best Industries Pd103	Meigooni et al.^(14)^	Monte Carlo simulation	Liquid water	0.670±0.020
		Measured, TLD	Solid water	0.690±0.055
		TG43U1	Liquid water	0.670±0.027
NAS MED3633 Pd103	Li et al.^(15)^	Monte Carlo simulation	Liquid water	0.677±0.020
		Measured, TLD	Solid water	0.680±0.041
		TG43U1	Liquid water	0.688±0.020

a $1U=1\;\text{cGy}•{\text{cm}}^{2}•h^{-1}$.

b Corrected per the TG‐43(U1) recommendations of the American Association of Physicists in Medicine.

**Figure 2:**
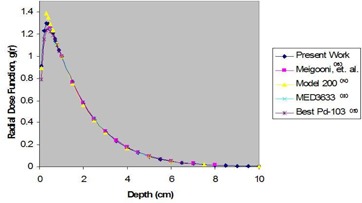
Comparison in water of the Monte Carlo–calculated radial dose function of the ADVANTAGE Pd103 source with radial dose functions of other commercially available sources.

The radial dose function of the source was calculated in water from 0.1 cm to 10 cm. The uncertainty of the calculated data is ±3%. Fig. 2 shows a comparison between the calculated *g*(*r*) of the ADVANTAGE source and a selection of other commercially available sources. Table 2 presents the values of *g*(*r*) in water. Those values were obtained by simulating the dose rate at each *g*(*r*) distance and then normalizing to the simulated dose rate at 1 cm. The result was then corrected for inverse square relation as per TG‐43.

**Table 2:** The calculated radial dose function of the ADVANTAGE Pd103 brachytherapy source in liquid water

	Radial dose function, g(*r*)			
	Meigooni *et al*.^(12)^			
Distance from source center *r* (cm)	Monte Carlo Liquid water	Measured TLD	Monte Carlo Solid water	Present work Monte Carlo Liquid water
0.1				0.915
0.2				1.234
0.3				1.296
0.4				1.290
0.5	1.263	1.243	1.289	1.260
0.6				1.213
0.7				1.160
0.75				1.134
0.8				1.106
0.9				1.053
1.0	1.000	1.000	1.000	1.000
1.5	0.761	0.720	0.750	0.768
2.0	0.579	0.536	0.555	0.576
2.5	0.431		0.406	0.429
3.0	0.323	0.296	0.292	0.318
3.5	0.235		0.211	0.233
4.0	0.177	0.157	0.153	0.173
4.5	0.127		0.107	0.127
5.0	0.092	0.085	0.077	0.092
5.5				0.069
6.0	0.050	0.048	0.042	0.050
6.5				0.037
7.0	0.029	0.030	0.023	0.028
7.5				0.020
8.0	0.018		0.014	0.015
8.5				0.011
9.0				0.008
9.5				0.006
10.0				0.005

Many treatment planning systems require a polynomial fit to *g*(*r*). For use of this data in those various treatment planning systems, the calculated *g*(*r*) in water in the range of 0.1 cm to 10 cm was fitted to a fifth‐order polynomial function defined as follows:

(3) $$g(r)=a_{0}+a_{1}r+a_{2}r^{2}+a_{3}r^{3}+a_{4}r^{4}+a_{5}r^{5}\;\;,$$

where $a_{0}=1.1983,\;a_{1}=7.3502E^{-2},\;a_{2}=-3.1789E^{-1},\;a_{3}=9.1913E^{-2},\;a_{4}=-9.9569E^{-3},\;\text{and}\;a_{5}=3.7557E^{-4}$. This fifth‐order polynomial fit has been found to fail to accurately reproduce the *g*(*r*) at radial distances greater that 10 cm; however, it is accurate at distances less than 10 cm.

The anisotropy function of the source was calculated in liquid water for distances ranging from 0.5 cm to 7 cm. Those values were obtained by simulating the dose rate at each angle and normalizing to the dose rate at 90 degrees and at the radial distance in question. The result was then corrected using the geometry function relationship as defined in TG‐43. The uncertainties of the calculated values range from ±5% to ±6%.

**Figure 3:**
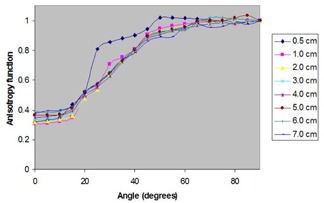
Variation of the Monte Carlo‐simulated *F*(*r*,θ) of the ADVANTAGE Pd103 source in liquid water at distances ranging from 0.5 cm to 7 cm.

**Figure 4:**
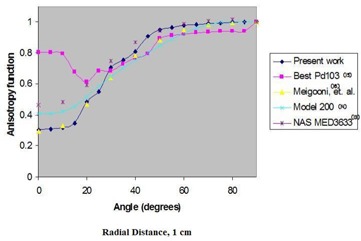
Comparison in liquid water at 1 cm radius of the Monte Carlo‐simulated anisotropy functions of the ADVANTAGE Pd103 source with the anisotropy functions of other commercially available sources.

**Figure 5:**
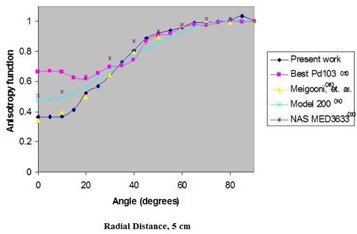
Comparison in liquid water at 5 cm radius of the Monte Carlo–simulated anisotropy function of the ADVANTAGE Pd103 source with the anisotropy functions of other commercially available sources.

Fig. 3 shows the variation of *F*(*r*,θ) in water as a function of distance from the source. Figs. 4 and 5 compare the anisotropy function of the ADVANTAGE source with those of several other commercially available sources at 1 cm and 5 cm respectively. From the anisotropy function of the ADVANTAGE source, the anisotropy factors $\varphi_{\text{an}}(r)$ and the anisotropy constant ${\bar{\varphi }}_{\text{an}}$ have been extracted. Table 3 shows the anisotropy functions, anisotropy factors, and anisotropy constant of the ADVANTAGE source in liquid water. Table 4 compares the anisotropy factors and constant with those of other commercially available sources. The calculated anisotropy constant for the ADVANTAGE source in water was 0.880±0.040.

**Table 3:** Monte Carlo–simulated two‐dimensional anisotropy function of the ADVANTAGE Pd103 brachytherapy source in liquid water

	*F*(*r*,θ)							
Angle θ (degrees)	0.5 cm	1.0 cm	2.0 cm	3.0 cm	4.0 cm	5.0 cm	6.0 cm	7.0 cm
0	0.319	0.307	0.320	0.337	0.349	0.365	0.392	0.379
5	0.333	0.310	0.324	0.340	0.356	0.365	0.384	0.397
10	0.349	0.321	0.335	0.352	0.367	0.371	0.390	0.399
15	0.436	0.350	0.362	0.381	0.395	0.413	0.425	0.430
20	0.520	0.482	0.482	0.490	0.500	0.522	0.521	0.518
25	0.807	0.549	0.539	0.544	0.553	0.568	0.579	0.577
30	0.852	0.705	0.641	0.627	0.626	0.641	0.647	0.650
35	0.880	0.752	0.724	0.724	0.716	0.727	0.741	0.740
40	0.902	0.807	0.796	0.794	0.792	0.802	0.805	0.782
45	0.941	0.906	0.895	0.882	0.873	0.890	0.882	0.858
50	1.021	0.948	0.926	0.912	0.903	0.921	0.918	0.887
55	1.020	0.964	0.946	0.936	0.930	0.940	0.937	0.888
60	1.016	0.976	0.963	0.951	0.946	0.957	0.936	0.956
65	1.009	0.984	0.975	0.967	0.966	0.992	0.990	0.975
70	1.003	0.990	0.986	0.979	0.976	0.992	1.018	0.953
75	0.997	0.994	0.994	0.988	0.986	0.997	1.019	0.961
80	0.992	0.997	0.999	0.993	0.975	1.013	1.009	0.985
85	0.990	0.999	1.000	0.999	0.989	1.033	1.005	0.973
90	1.000	1.000	1.000	1.000	1.000	1.000	1.000	1.000
$\varphi_{\text{an}}(r)$	0.940	0.890	0.860	0.860	0.870	0.860	0.880	0.870
${\bar{\varphi }}_{\text{an}}$	0.880±0.040							

**Table 4:** Comparison of the Monte Carlo–simulated anisotropy factors and constant of the ADVANTAGE Pd103 source and other commercially available sources in liquid water

	1D Anisotropy function, $\varphi_{\text{an}}(r)$				
Radial distance (cm)	Present work	Meigooni et al.^(12)^	Model 200 Williamson^(13)^	Best Pd103 Meigooni et al.^(14)^	NAS MED3633 Li et al.^(15)^
0.5	0.94	0.94	0.89		
1.0	0.89	0.88	0.87	0.90	0.93
2.0	0.86	0.86	0.86	0.87	0.92
3.0	0.86	0.85	0.87	0.88	0.92
4.0	0.87	0.85	0.87	0.88	0.93
5.0	0.86	0.88	0.87	0.89	0.92
6.0	0.88				
7.0	0.87				
${\bar{\varphi }}_{\text{an}}$	0.88	0.88	0.87	0.88	0.92

## IV. DISCUSSION AND CONCLUSIONS

The dose rate constant of the ADVANTAGE Pd103 source was found to be $0.709\pm 0.014\;\text{cGy}•h^{-1}•U^{-1}$. This value is in good agreement with other commercially available Pd103 sources. Table 1 compares the dose rate constant of the ADVANTAGE source with the dose rate constants calculated for several other commercially available Pd103 sources such as the Model 200 source, by Williamson^(13)^; previous work on the ADVANTAGE source calculated by Meigooni et al.^(12)^; the NAS MED3633 calculated by Li and Palta^(15)^; and the Best Pd103 calculated by Meigooni et al.^(14)^

Fig. 2 compares the *g*(*r*) for the new source with those for other commercially available sources such as the Model 200 source determined by Williamson,^(13)^ previous work on the ADVANTAGE source determined by Meigooni et al.,^(12)^ NAS MED3633 determined by Li and Palta,^(15)^ and the Best Pd103 determined by Meigooni et al.^(14)^ The figure shows that the radial dose function of the ADVANTAGE Pd103 source is in good agreement with those of the other sources.

Fig. 3 shows the variation of the anisotropy function with radial distance in water. The *F*(*r*, θ) of the ADVANTAGE source in water was compared with those of the Model 200 source determined by Weaver,^(16)^ previous work on the ADVANTAGE source determined by Meigooni et al.,^(12)^ the NAS MED3633 determined by Li and Palta,^(15)^ and the Best Pd103 determined by Meigooni et al.^(14)^ (Figs. 4 and 5). The figures show good agreement between the ADVANTAGE source and other Pd103 sources for angular ranges of 20 degrees to 90 degrees. Below 20 degrees, differences in endcap construction yield significant deviations in the plotted anisotropy functions.

Table 3 presents the values of the measured and calculated anisotropy functions, anisotropy factors, and anisotropy constants for the ADVANTAGE source in liquid water. Table 4 compares the anisotropy factors and constant of the ADVANTAGE source with other commercially available Pd103 sources. The anisotropy constant of the ADVANTAGE Pd103 source in water was found to be 0.880±0.040.

The dosimetric characteristics of the ADVANTAGE source were theoretically determined based on TG‐43(U1) recommendations. These characteristics were found to be comparable to the values reported for other commercially available Pd103 sources. As per TG‐43(U1), the parameters determined in liquid water are recommended for clinical applications.

## ACKNOWLEDGMENT

This project was supported by IsoAid LLC. Special thanks are due to Ali Meigooni for his assistance in the preparation of this manuscript.
